# Association Between the Mandibular Inferior Cortical Shape on Panoramic Radiographs and the Prognosis of Hydroxyapatite Implant Placement

**DOI:** 10.7759/cureus.83240

**Published:** 2025-04-30

**Authors:** Noriyuki Sugino, Yutaka Kitamura, Akihiro Kuroiwa, Hiroko Kuroiwa, Nanae Dewake, Keiichi Uchida, Yuji Kurihara, Nobuo Yoshinari, Nobuyuki Udagawa, Akira Taguchi

**Affiliations:** 1 Department of Oral and Maxillofacial Radiology, School of Dentistry, Matsumoto Dental University, Shiojiri, JPN; 2 Department of Oral and Maxillofacial Surgery and Dental Implant, Center of Oral and Maxillofacial Surgery and Dental Implant in Shinshu, Obuse, JPN; 3 Department of Dental Materials and Science, School of Dentistry, Matsumoto Dental University, Shiojiri, JPN; 4 Department of Pediatric Dentistry, School of Dentistry, Matsumoto Dental University, Shiojiri, JPN; 5 Department of Operative Dentistry, Endodontology and Periodontology, School of Dentistry, Matsumoto Dental University, Shiojiri, JPN; 6 Department of Oral Diagnostics and Comprehensive Dentistry, Matsumoto Dental University Hospital, Shiojiri, JPN; 7 Department of Oral and Maxillofacial Surgery, School of Dentistry, Matsumoto Dental University, Shiojiri, JPN; 8 Department of Biochemistry, School of Dentistry, Matsumoto Dental University, Shiojiri, JPN

**Keywords:** hydroxyapatite (ha) implant, implant prognosis, mandibular cortical index (mci), mandibular cortical width (mcw), mandibular inferior cortical shape, panoramic radiograph

## Abstract

Objective

Dental implant therapy is a widely accepted treatment option for edentulous patients. However, implant success can be influenced by various factors, including systemic bone conditions such as osteoporosis. This study aimed to evaluate the association between the mandibular inferior cortical shape observed on panoramic radiographs and the prognosis of hydroxyapatite (HA)-coated implant placement in the maxilla.

Materials and methods

A total of 125 patients (381 maxillary implants) who underwent HA implant placement with a minimum five-year follow-up were included. The mandibular inferior cortical shape was classified using panoramic radiographs based on the mandibular cortical width (MCW) and mandibular cortical index (MCI). Preoperative trabecular bone CT values were measured using Simplant® software. Logistic regression analysis was performed to investigate the relationship between cortical shape, trabecular bone CT values, and implant prognosis.

Results

The overall implant survival rate was 93.2%. Patients with mildly to moderately or severely eroded mandibular cortices had a significantly higher risk of implant failure compared to those with normal cortices (OR = 13.50, p = 0.028). In cases with normal cortices, an increase of 100 HU in trabecular bone CT values was associated with a higher risk of implant failure (OR = 1.50, p = 0.003). Implant survival was positively associated with the number of remaining teeth, while implants placed in the maxillary posterior region tended to show lower survival.

Conclusion

Mandibular inferior cortical shape assessed via panoramic radiographs is significantly associated with the prognosis of HA-coated implant placement. Panoramic radiograph-based evaluation may serve as a useful screening tool for identifying patients at increased risk of implant failure and for supporting individualized treatment planning, particularly in elderly or osteoporotic populations.

## Introduction

Osteoporosis is a systemic skeletal disorder characterized by reduced bone strength due to aging or hormonal decline in women, resulting in an increased risk of fractures [1,2]. Since osteoporosis often progresses without noticeable symptoms, it is typically diagnosed only after a fracture has occurred. Moreover, fractures associated with osteoporosis can trigger a “fracture cascade,” whereby one fracture increases the likelihood of subsequent ones [3]. This phenomenon significantly impacts public health by reducing the quality of life (QOL) in older adults and increasing mortality rates.

Dual-energy X-ray absorptiometry (DXA) is the most widely used method for osteoporosis screening and diagnosis [4,5]. However, the screening rate in Japan remains low, with a national average of approximately 5.4% [6,7]. Since asymptomatic patients at high risk of fractures are unlikely to seek medical care, alternative screening approaches are needed. In recent years, panoramic radiographs have gained attention as a non-invasive tool for assessing the mandibular inferior cortical shape to facilitate early detection of osteoporosis [8,9]. As panoramic radiographs are routinely taken in dental clinics, this approach places minimal burden on patients. Specifically, the presence of discontinuities along the endosteal surface of the mandibular cortex, along with cortical thinning, has been reported to reflect the progression of osteoporosis.

Bone strength depends on both bone mineral density (BMD) and bone quality, with approximately 70% of strength attributed to BMD and the remaining 30% to bone quality [2]. Bone quality includes several factors, such as trabecular microarchitecture, bone turnover, microdamage accumulation, and mineralization. While BMD is typically measured using DXA, quantitative computed tomography (QCT), microdensitometry (MD), or quantitative ultrasound (QUS), bone quality is assessed using parameters such as trabecular bone score (TBS), bone turnover markers, and serum levels of vitamins D and K. Although panoramic radiographs do not provide a direct quantitative measure of BMD or bone quality, the degree of cortical resorption in the mandibular inferior border can be visually evaluated and may serve as an indirect indicator of both.

Osteoporosis is also considered a local risk factor in dental implant treatment [10,11]. Patients with osteoporosis tend to exhibit reduced bone strength and delayed bone remodeling, which may compromise the initial stability of implants, hinder osseointegration, and lead to marginal alveolar bone loss [11,12]. However, current evidence regarding the long-term prognosis of implants in osteoporotic patients is limited, and further studies are needed to clarify the relationship between osteoporosis screening indicators and implant treatment outcomes.

Therefore, this study aimed to investigate the association between mandibular inferior cortical shape assessed on panoramic radiographs and the prognosis of hydroxyapatite (HA)-coated implant placement in the maxilla. Maxillary implants were exclusively analyzed because mandibular implants in our initial cohort showed an extremely low failure rate, limiting the feasibility of statistical evaluation. Clarifying these relationships may lead to the development of simple and effective preoperative screening strategies to improve implant outcomes, particularly in elderly or osteoporotic populations.

## Materials and methods

Study participants

This retrospective cohort study included 125 Japanese patients (42 males and 83 females), aged 26-83 years, who underwent maxillary implant placement at the Center of Oral and Maxillofacial Surgery and Dental Implant in Shinshu between 2008 and 2012. A total of 381 maxillary implants were analyzed. All patients had a minimum follow-up period of five years. Initially, both maxillary and mandibular implants were considered for analysis. However, among the 285 mandibular implant cases during the study period, only one failure occurred. To ensure statistical reliability and proper interpretation of the analysis, mandibular implants were excluded from the study. All patients who met the inclusion criteria during the study period were consecutively enrolled. No selection based on general health conditions or comorbidities was performed; therefore, the sample reflects the actual patient population treated during that time. Patients who required bone augmentation procedures - such as bone grafting, guided bone regeneration (GBR), or maxillary sinus floor elevation - due to severe bone resorption were excluded. Cases involving immediate implant placement following tooth extraction were also excluded. Additionally, patients who received removable prostheses such as implant-supported overdentures were excluded, as this study focused exclusively on fixed prosthetic restorations.

This study was approved by the Ethics Committee of Matsumoto Dental University (approval no. 0281). Written informed consent was obtained from all participants prior to enrollment. The study was conducted in accordance with ethical principles, with full respect for confidentiality and voluntary participation. All collected data were anonymized, and participants were informed of their right to withdraw from the study at any point without any adverse consequences.

Imaging examinations

Panoramic radiographs were obtained using the AZ3000 unit (Asahi Roentgen Co., Ltd., Kyoto, Japan) following the standard hospital protocol, which included a tube voltage of 64.0 kV, a tube current of 8.0 mA, and an exposure time of 12.0 seconds. The images were processed using a computed radiography system (Regius Model 170; Konica Minolta Japan, Tokyo, Japan) and evaluated on a diagnostic monitor (PGL21; WIDE Corporation, Korea).

Multi-detector computed tomography (MDCT) scans were performed using the Emotion 16® system (Siemens Healthineers Japan, Tokyo, Japan) according to hospital protocols, with scanning parameters set at 130.0 kV and 160.0 mA. During scanning, a diagnostic stent was placed in the oral cavity. Image reconstruction was conducted using a slice thickness of 0.75 mm and a slice interval of 0.20 mm. The DICOM data were then imported into the Simplant® software (Dentsply Sirona, Tokyo, Japan) for further analysis.

Implant placement procedure

HA-coated implants with high biocompatibility (Spline Twist™ MP-1®; Zimmer Biomet Dental, Palm Beach Gardens, FL) were used in this study [13,14]. The available options included diameters of 3.25 mm, 3.75 mm, and 5.0 mm and lengths of 8 mm, 10 mm, 11.5 mm, 13 mm, and 15 mm. The most appropriate combination of implant diameter and length was selected based on the patient’s alveolar bone dimensions using the Simplant® planning system. All surgeries were performed by a single oral surgeon with 33 years of clinical experience (since 2008). Surgical stents were designed based on Simplant® planning data and used during surgery to ensure optimal positioning, angulation, and depth of implant placement. In total, 381 implants were placed, with an average of 3.0 implants per patient.

Postoperative prosthetic restoration was also performed by the same surgeon. Only fixed prosthetic restorations (single crowns or fixed partial dentures) were included in this study. Removable prostheses such as overdentures were excluded to ensure homogeneity and analytical consistency. Cement-retained restorations were primarily used, except in cases where screw-retained restorations were clinically indicated.

Evaluation methods

Classification of Mandibular Inferior Cortical Shape

Preoperative evaluation of the mandibular inferior cortical shape was independently conducted by a board-certified oral radiologist using panoramic radiographs. The classification was based on a combination of the mandibular cortical width (MCW) and the mandibular cortical index (MCI,) as previously described [8,9].

**Figure 1:**
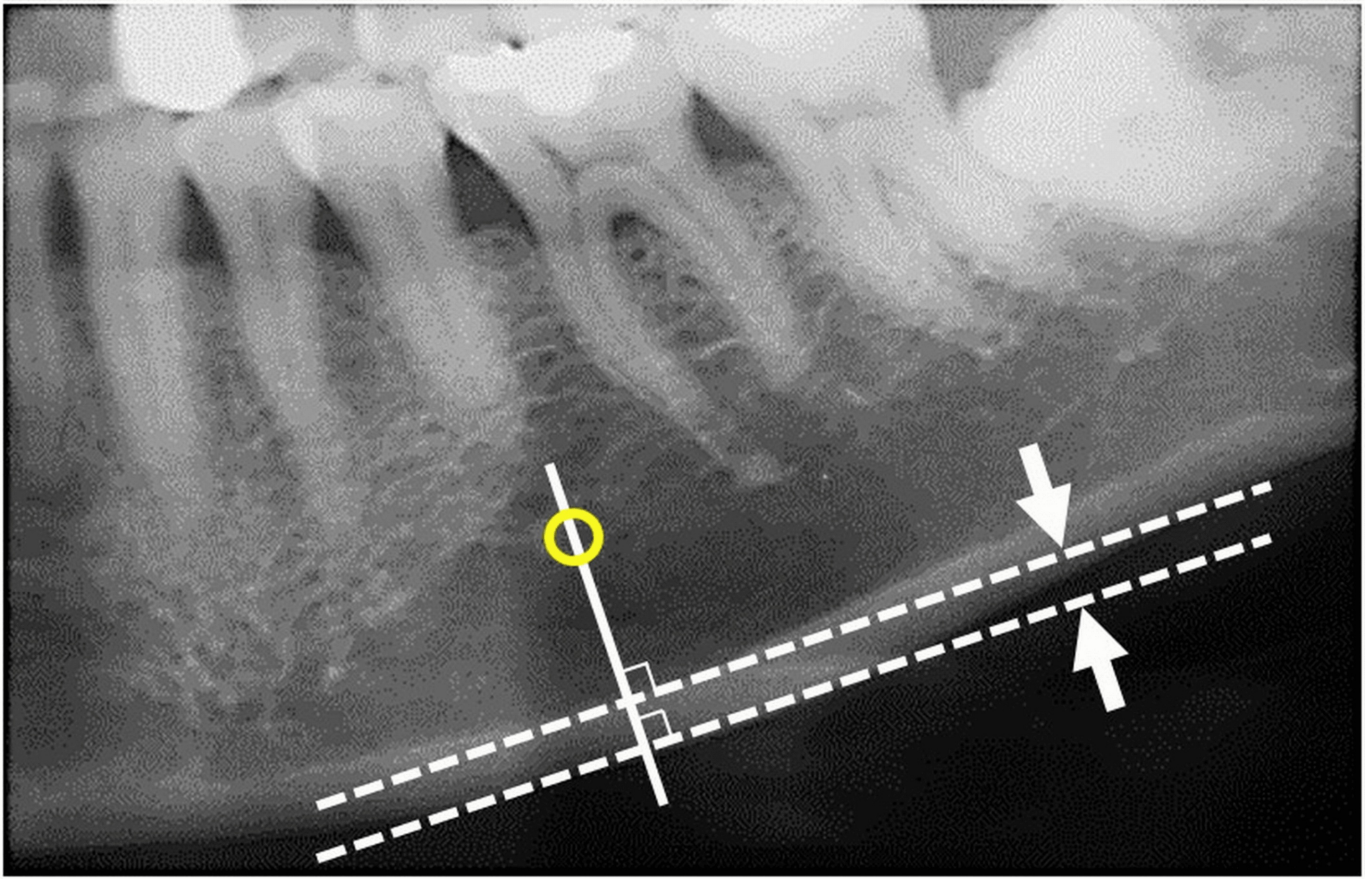
Measurement of the mandibular cortical width (MCW) The MCW was measured bilaterally in the mental foramen region. A line parallel to the long axis of the mandible and tangential to the inferior border was drawn (dashed lines), and a perpendicular line intersecting the inferior border of the mental foramen was constructed (solid line). The cortical width (indicated by the double-headed arrow) was measured along this perpendicular line. The average of both sides was used for the analysis. The yellow circle denotes the mental foramen.

The MCW was measured bilaterally at the region of the mental foramen (Figure 1). A line was drawn parallel to the long axis of the mandible and tangential to the inferior border. A perpendicular line intersecting the inferior border at the level of the mental foramen was then constructed, and the cortical width was measured along this line. The mean value of both sides was used for analysis. In this study, we adopted a cutoff value of 3.0 mm, which has been reported as the average threshold for identifying individuals at higher risk of osteoporosis [9].

**Figure 2:**
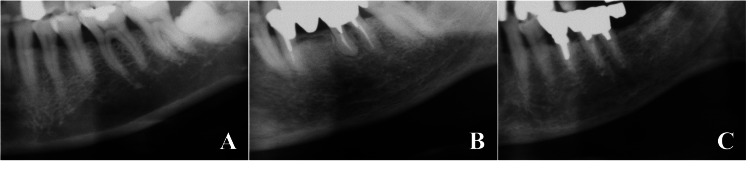
Classification of the mandibular cortical index (MCI) (A) Normal cortex: The endosteal margin of the cortex is even and sharp bilaterally. (B) Mildly to moderately eroded cortex: The endosteal margin shows semilunar defects or exhibits endosteal cortical residues. (C) Severely eroded cortex: The cortical layer shows extensive endosteal cortical residues and appears clearly porous.

The MCI was assessed by examining the mandibular region distal to the mental foramen on both sides, and the side with the most severe cortical erosion was used for classification (Figure 2). The cortical shape was categorized into three types: normal cortex, in which the endosteal margin was smooth and clearly defined on both sides; mildly to moderately eroded cortex, where the endosteal margin showed semilunar defects or endosteal cortical residues; and severely eroded cortex, characterized by extensive endosteal cortical residues and a porous appearance.

Measurement of Preoperative Trabecular Bone CT Values

**Figure 3:**
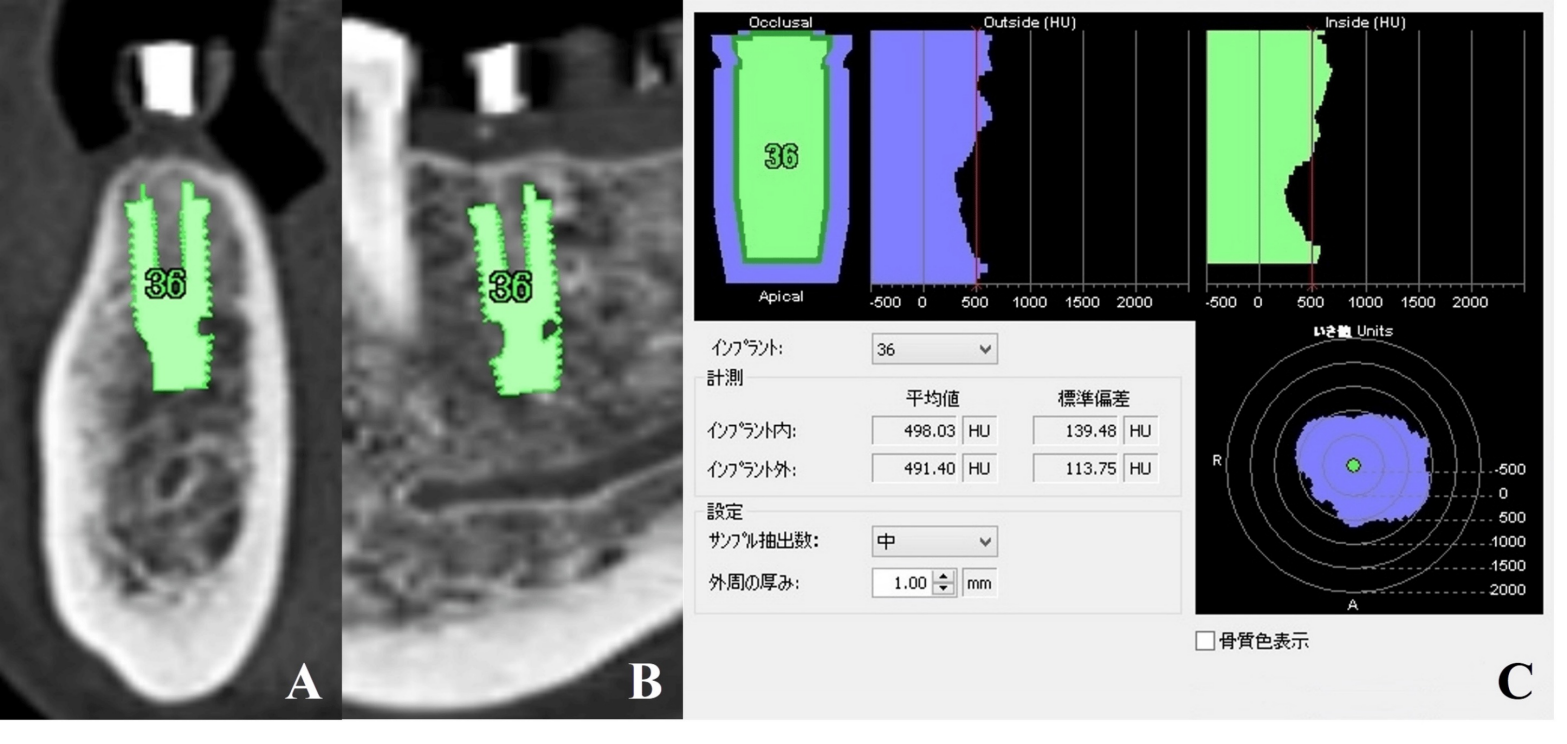
Measurement of preoperative trabecular bone CT values Preoperative trabecular bone CT values were measured using the implant simulation software (Simplant®). The implant was virtually placed at the planned site within Simplant® (A: cross-sectional image; B: panoramic image), and CT values were automatically calculated in the inner and outer regions of the implant body (C: measurement results).

Preoperative trabecular bone CT values were measured using Simplant®. The software automatically calculated CT values in the inner and outer regions of the planned implant sites. These values were recorded by the surgeon based on preoperative imaging data obtained during planning (Figure 3).

Assessment of Implant Prognosis

Implant prognosis was evaluated using established clinical criteria [15]. Success was defined by the absence of mobility, functional and esthetic stability of the implant-supported prosthesis, and the lack of pain, discomfort, sensory disturbances, or signs of infection. In addition, marginal alveolar bone loss was evaluated to assess peri-implant bone stability. Although vertical bone loss of ≤0.2 mm per year after loading is generally considered within the normal range [15], this study defined peri-implantitis-associated bone loss as ≤3 mm from the time of implant placement [16]. Marginal bone levels were measured using intraoral radiographs taken immediately after implant placement, focusing on the mesial and distal aspects of each implant. For further analysis, the implant sites were classified into two anatomical categories: the maxillary anterior region (canine to canine) and the maxillary posterior region (first premolar to second molar), as defined in previous studies [17,18].

Statistical analysis

Although a range of implant diameters and lengths were used based on individual clinical conditions, these variables were not included in the analysis, as previous research has shown that they have minimal impact on implant survival rates [19-22].

Logistic regression analysis was performed to evaluate factors associated with implant prognosis. The dependent variable was implant outcome (success or failure), while the independent variables included age, sex, implant placement site, mandibular inferior cortical shape, preoperative trabecular bone CT values, number of remaining natural teeth, and the presence of diabetes or hypertension. Dummy variables were assigned for categorical variables, such as sex (female = 0, male = 1), implant site (anterior = 0, posterior = 1), cortical shape (normal = 0, eroded = 1), diabetes (absent = 0, present = 1), and hypertension (absent = 0, present = 1). Preoperative trabecular bone CT values were evaluated per 100 Hounsfield units (HU), and the number of remaining teeth was treated as a continuous variable. These variable definitions were based on standard statistical modeling practices. Categorical variables were converted to dummy variables, and continuous measures such as residual teeth count and CT values were analyzed accordingly. Given the retrospective nature of the study, no prior sample size calculation was performed. All eligible cases during the study period were included in the analysis.

To assess potential interaction effects between mandibular cortical shape and CT values, two interaction terms were introduced: z1 = c1 × ct100 and z2 = c0 × ct100, enabling separate evaluation of CT value effects by cortical shape group. Model selection was based on the Akaike Information Criterion (AIC), and non-significant variables were excluded from the final model.

All statistical analyses were conducted using R (version 4.0.5; The R Foundation for Statistical Computing, Vienna, Austria). A significance level of p < 0.05 was considered statistically significant.

## Results

The mean age of participants was 59.3 ± 8.7 years, with a mean observation period of 7.3 ± 1.6 years and mean preoperative trabecular bone CT values of 485.5 ± 270.9 HU (Table 1). Among the 381 implants analyzed, 26 cases resulted in implant failure. The survival rates were 95.8% for implants placed in the maxillary anterior region, 91.6% for those in the posterior region, and 93.2% overall.

**Table 1:** Characteristic of the study participants SD: standard deviation; HU: Hounsfield units

Variables	Mean ± SD or Number of subjects (implants)
Age (years)	59.3 ± 8.7
Sex	
Female	83
Male	42
Number of HA implant placements	381
Observation period (months)	87.8 ± 19.2
Number of HA implant failures	26
Classification of mandibular inferior cortical shape	
Normal cortex	69
Mildly to moderately eroded cortex	42
Severely eroded cortex	14
Preoperative trabecular bone CT values	485.5 ± 270.9HU
Number of remaining natural teeth	18.4 ± 6.6
Diabetes	5 (17)
Hypertension	25 (85)

The distribution of mandibular inferior cortical shape classifications included 69 patients with a normal cortex, 42 with a mildly to moderately eroded cortex, and 14 with a severely eroded cortex. When comparing implant failure rates by placement site, the odds ratio (OR) for failure in the maxillary anterior region relative to the posterior region was 2.65 (95% confidence interval (CI): 0.99-7.09; p = 0.053) (Table 2). The OR for implant failure in patients with a mildly to moderately or severely eroded cortex, compared to those with a normal cortex, was 13.50 (95% CI: 1.32-138.00; p = 0.028), indicating a statistically significant association between cortical erosion and implant failure.

**Table 2:** Logistic regression analysis for the predictors of implant failure AIC (Akaike Information Criterion) = 180.39; Implant placement site (s): the maxillary anterior region (0), the maxillary posterior region (1); Mandibular inferior cortical shape (c): normal cortex (0), mildly to moderately eroded or severely eroded cortex (1); Preoperative trabecular bone CT values (ct100): z1 = 𝑐1 · ct100, z2 = 𝑐0 · ct100; Number of remaining natural teeth (nteeth)

Variables	Odds ratio	95% CI (Lower)	95% CI (Upper)	p-value
s1	2.65	0.99	7.09	0.053
c1	13.50	1.32	138.00	0.028
z1	1.08	0.87	1.34	0.482
z2	1.50	1.15	1.95	0.003
nteeth	0.93	0.87	0.99	0.017

**Figure 4:**
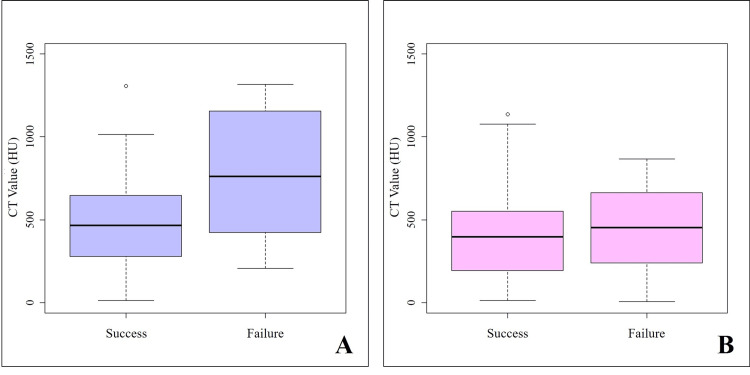
Relationship between the mandibular inferior cortical shape and implant prognosis according to preoperative CT values A: Normal cortex. B: Mildly to moderately eroded cortex and severely eroded cortex. Among patients with a normal cortex, each 100-HU increase in preoperative trabecular bone CT values was associated with a 1.50-fold increase in the odds of implant failure (95% CI: 1.15–1.95; p = 0.003).

Among patients with a normal cortex, each 100 HU increase in preoperative trabecular bone CT values was associated with a 1.50-fold increase in the odds of implant failure (95% CI: 1.15-1.95; p = 0.003) (Figure 4). This finding suggests a significant interaction between cortical shape classification and CT values at the implant sites. Furthermore, the number of remaining natural teeth was inversely associated with implant failure. Each additional remaining tooth reduced the odds of failure, with an OR of 0.93 (95% CI: 0.87-0.99; p = 0.017), indicating a protective effect of dentition status on implant survival. Although diabetes and hypertension were included as explanatory variables in the initial logistic regression model, neither showed a significant association with implant prognosis (p > 0.05) and were therefore excluded from the final model.

## Discussion

This study demonstrated that a mandibular inferior cortical shape classified as mildly to moderately eroded or severely eroded was significantly associated with an increased risk of implant failure. This finding is consistent with previous reports [11,12] and suggests that, compared to a normal cortex, cortical erosion may negatively affect both the initial stability and long-term maintenance of implants due to reduced bone strength and impaired bone remodeling. In particular, severely eroded cortices - characterized by cortical discontinuities and pronounced linear resorption - may indicate more severe deterioration of bone quality, thereby exerting a greater influence on implant success rates. Although DXA is widely used for diagnosing osteoporosis [4,5], its use in dental clinics is often impractical. In contrast, panoramic radiographs, which are routinely obtained in dental practice, provide a promising alternative for assessing mandibular cortical shape. This approach allows for early identification of osteoporosis in patients undergoing implant therapy and supports individualized treatment planning and postoperative management based on risk stratification.

A significant association was also observed between mandibular inferior cortical shape and preoperative trabecular bone CT values. Among patients with a normal cortex, higher preoperative CT values were strongly correlated with an increased risk of implant failure. Although high bone density is generally thought to enhance initial implant stability, excessively dense bone may reduce blood supply and impair bone remodeling, ultimately compromising long-term implant success. Moreover, extremely dense bone can increase the risk of thermal injury during implant placement due to excessive frictional heat. It has been reported that irreversible bone necrosis can occur when bone temperature exceeds 53°C, and exposure to temperatures above 47°C for more than one minute may lead to thermal osteonecrosis [23,24]. Additionally, high insertion torque may induce compressive necrosis by impairing local microcirculation and damaging osteocytes. Dense cortical bone also has low elasticity, which can lead to stress concentration and an increased risk of fracture [25]. When preoperative trabecular bone CT values are abnormally high, clinicians should carefully consider drill selection, drilling speed, irrigation volume, and insertion torque to preserve blood supply and promote favorable bone remodeling during implant placement.

This study also found that implants placed in the maxillary posterior region were associated with 2.65 times greater odds of failure compared to those in the anterior region. This finding is consistent with previous reports [26,27], which suggest that the maxillary posterior region presents a higher risk of implant failure. However, the literature contains conflicting results regarding the prognosis of implants in the anterior versus posterior maxilla. Some studies have reported a higher risk in the anterior region [21], while others have found no significant difference between the two sites [28]. Several factors may contribute to the increased risk in the posterior maxilla, including higher occlusal forces and greater stress concentration on implants. In addition, proximity to the maxillary sinus often leads to reduced bone volume, requiring bone grafting or sinus lift procedures that may further influence outcomes. Although the maxillary anterior region has thinner cortical bone, making it more difficult to achieve primary stability, the reduced occlusal load in this area may mitigate long-term failure risk. Variability in study populations (e.g., age distribution and systemic health), research design, prosthetic strategy (e.g., bridge vs. single crown), timing of implant placement (e.g., immediate vs. delayed), implant system, and operator experience may all contribute to inconsistent findings. Future research should prioritize long-term, multicenter studies to clarify site-specific risk factors and improve predictive models for implant success.

Additionally, this study revealed that a higher number of remaining natural teeth exerted a protective effect on implant survival. Each additional tooth was associated with a 7% reduction in the risk of implant failure (OR = 0.93). This suggests that natural dentition helps distribute occlusal forces more evenly, reducing the burden placed on implants. Li et al. [29] reported that implant failure tends to be higher in areas subjected to excessive occlusal loading. In edentulous patients, who rely solely on implants for occlusal support, appropriate prosthetic design and occlusal management are critical to minimizing excessive force and improving long-term outcomes.

This study has several limitations. First, the study population was limited to patients treated at a single dental institution by a single clinician, which may restrict the generalizability of the findings. Second, the sample size was relatively small, and only one case of mandibular implant failure was recorded, limiting the statistical power to evaluate mandibular implant outcomes. Third, all implants used in this study were HA-coated and known for their high biocompatibility [13,14]; therefore, the results may not be applicable to other implant systems. Fourth, this study included only cases with fixed prosthetic restorations; patients who received removable prostheses were excluded to ensure consistency in implant prognosis evaluation. Fifth, information on the use of bisphosphonate medications - a known risk factor for implant complications - was not collected in this retrospective study. As a result, the potential influence of bisphosphonate therapy on implant prognosis could not be assessed. Future prospective studies should incorporate this variable to improve the comprehensiveness of risk evaluation. Furthermore, the assessment of panoramic radiographs was based on visual evaluation, which may introduce observer bias. Incorporating more objective approaches, such as artificial intelligence-based image analysis, may improve diagnostic accuracy and reproducibility. To further validate the clinical utility of panoramic radiograph-based screening, future studies should involve multicenter cohorts with long-term follow-up and randomized controlled trials (RCTs) to establish more definitive conclusions.

## Conclusions

This study demonstrated a significant association between mandibular inferior cortical shape, as evaluated using panoramic radiographs, and the prognosis of maxillary implant treatment. A mildly to moderately eroded or severely eroded cortex, along with elevated preoperative trabecular bone CT values, was associated with an increased risk of implant failure - likely due to impaired bone remodeling and reduced vascularity. Implants placed in the maxillary posterior region also showed a higher failure rate, possibly due to increased occlusal loading and reduced bone volume. Conversely, a greater number of remaining natural teeth was associated with improved implant survival, suggesting a protective role of natural dentition in distributing occlusal forces.

These findings indicate that panoramic radiographic assessment of mandibular inferior cortical shape could serve as a valuable tool for preoperative risk evaluation in implant therapy. This approach may facilitate the early detection of osteoporosis and enable individualized treatment planning and postoperative management based on patient-specific risk factors. Further multicenter studies with long-term follow-up are warranted to validate these findings and establish robust predictive indicators for enhancing the success of implant therapy.
